# California sea lions employ task-specific strategies for active touch sensing

**DOI:** 10.1242/jeb.243085

**Published:** 2021-11-05

**Authors:** Alyx O. Milne, Llwyd Orton, Charlotte H. Black, Gary C. Jones, Matthew Sullivan, Robyn A. Grant

**Affiliations:** 1 Faculty of Science and Engineering, Manchester Metropolitan University, Chester Street, Manchester, M1 5GD, UK; 2 Events Team, Blackpool Zoo, East Park Drive, Blackpool, FY3 8PP, UK

**Keywords:** Whiskers, Sensorimotor, Haptics, Tactile, Pinnipeds

## Abstract

Active sensing is the process of moving sensors to extract task-specific information. Whisker touch is often referred to as an active sensory system as whiskers are moved with purposeful control. Even though whisker movements are found in many species, it is unknown whether any animal can make task-specific movements with their whiskers. California sea lions (*Zalophus californianus*) make large, purposeful whisker movements and are capable of performing many whisker-related discrimination tasks. Therefore, California sea lions are an ideal species to explore the active nature of whisker touch sensing. Here, we show that California sea lions can make task-specific whisker movements. California sea lions move their whiskers with large amplitudes around object edges to judge size, make smaller, lateral stroking movements to judge texture and make very small whisker movements during a visual task. These findings, combined with the ease of training mammals and measuring whisker movements, makes whiskers an ideal system for studying mammalian perception, cognition and motor control.

## Abstract (precis)

**Highlighted Article:** California sea lions engage in task-specific active touch sensing with their whiskers to discriminate size and differentiate textures, indicating that their whiskers are truly an active sensory system.

## INTRODUCTION

A key component of sensing is the ability to move sensors so as to extract task-specific information – a process referred to as active sensing (Prescott et al., 2011; Yang et al., 2016). Focusing sensors towards salient features within an environment allows quick and accurate identification of object attributes and location (Klatzky et al., 1987). Indeed, sensory perception is modulated by a range of factors, including attention, cognitive load, kinaesthesia and experience (Heller and Myers, 1983; Klatzky et al., 1987). For many senses, such as somatosensation, performance can be improved across sensory tasks by making precise and specific movements of the sensor (Gibson, 1962; Heller and Myers, 1983; Klatzky et al., 1987). Human fingertips are an active sensory system as they make purposeful, task-specific movements, such as lateral movements to determine object texture and vertical movements to judge object softness (Gibson, 1962; Lederman and Klatzky, 1987). Rather than fingertips, the primary tactile sensory system in many mammals is the whisker system. Whiskers are touch-sensitive facial hairs that are only truly absent in humans, great apes, rhinoceros and some species of cetaceans (Beddard, 1902; Evans et al., 2019; Muchlinski et al., 2013, 2020; Grant and Goss, 2021). Whiskers are actively controlled in many species (Grant et al., 2018; Muchlinski et al., 2020) via a specialised network of intrinsic muscles that are conserved from marsupials to primates (Grant et al., 2013a; Muchlinski et al., 2013).

While many studies have referred to whiskers as an active sensory system (Grant and Arkley, 2016; Grant et al., 2009; Prescott et al., 2011), no previous studies have quantitatively measured task-specific whisker movements in any animal. There have been many studies that have quantified whisker movement strategies (Carvell and Simons, 1995, 1996; Dehnhardt and Dücker, 1996; Towal and Hartmann 2006; Grant et al., 2009; Arkley et al., 2014; Schroeder and Ritt, 2016). However, this is the first study to document differences that depend on two behavioural contingencies presented to the same animal, suggesting that changes in whisker kinematics reflect a change in sensory goals on the part of the animal. Carvell and Simons (1995) were the first to identify that animals may make task-specific whisker movements. They found that whisker angles and the frequency of whisker movements varied in rats trained to discriminate between finely textured surfaces and those trained to differentiate between widely spaced textured surfaces. However, different individuals undertook each texture task. Dehnhardt and Dücker (1996) were the first to document that California sea lions (*Zalophus californianus*) adopted task-specific exploratory strategies. Specifically, during a shape discrimination task, the sea lion's head movements appeared to follow the contour of a shape (Dehnhardt and Dücker, 1996). However, these were only qualitative descriptions and the sea lion head and whisker movements were not explicitly measured. Certainly, as one of the most specialised sensory systems (Prescott et al., 2011), the mammalian whisker system is a likely candidate for finding evidence of task-specific active sensing, especially in California sea lions.

Pinnipeds, including seals, sea lions and walruses, have the most prominent and sensitive whiskers of any mammal (Dehnhardt et al., 1998; Dykes, 1975; Hyvarinen, 1989; Marshall et al., 2006). Compared with other pinnipeds, California sea lions move their whiskers with larger amplitudes (Milne et al., 2020) and can orient them towards moving objects (Milne and Grant, 2014; Milne et al., 2020). California sea lions can also use their whiskers to discriminate between different object shapes and sizes (Dehnhardt, 1990, 1994; Dehnhardt and Dücker, 1996) with the same sensitivity as human fingertips. Therefore, California sea lions are an ideal species to further explore the active nature of whisker touch sensing. Here, we investigated whether California sea lions can make task-specific whisker movements by measuring their head and whisker movements during three different discrimination tasks. Evidence of this would provide the first quantitative description of task-specific control of a tactile sensory system in any animal.

We trained a female California sea lion, named Lo, to sequentially complete texture, size and visual brightness discrimination tasks. The tasks required Lo to find one target stimulus amongst two distractor stimuli. If Lo were to adopt task-specific whisker movements, we would expect her whisker movements and positions to differ between the discrimination tasks, as Lo would focus on different stimuli features in order to efficiently complete each task.

## MATERIALS AND METHODS

One female California sea lion, *Zalophus californianus* (Lesson 1828) (Lo, aged 15 years) completed all aspects of training and reached the threshold required for data collection (see Fig. S1 for training details). Four California sea lions were originally trained; however, during training, two were moved to another collection and one refused to wear the blindfold and had a prominent right-hand bias, so did not perform to an appropriate threshold level. All procedures were carried out in accordance with Manchester Metropolitan University ethics regulations and approved by the local ethics committee at Blackpool Zoo.

### Apparatus

**Fig. 1.**
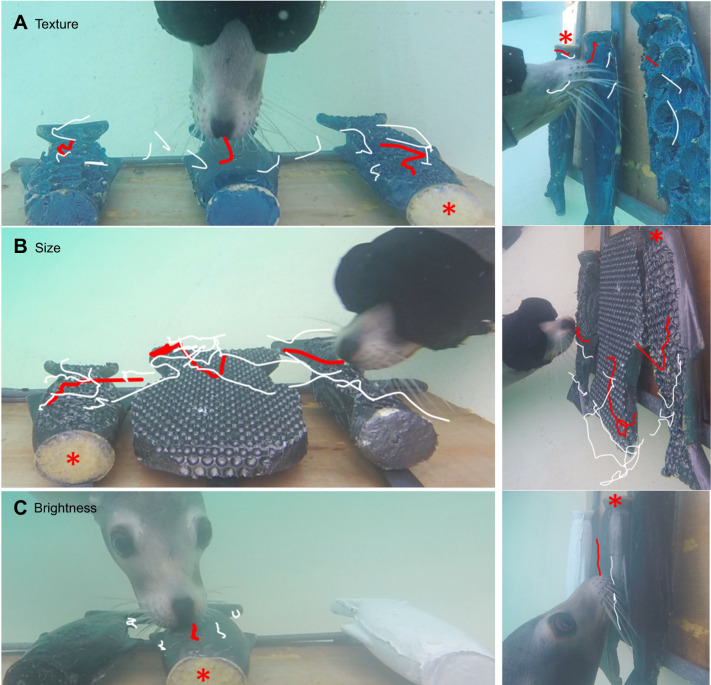
**Experimental setup.** (A–C) Example whisker (white) and head (red) traces in the top-down (left) and side-on (right) video views of Lo the California sea lion performing the texture (A), size (B) and brightness (C) discrimination tasks. A point on the whisker shafts was tracked to indicate whisker movement. The whiskers and head moved the most during the shape task, less on the texture task and the least on the visual brightness task. The different stimuli can also be seen, with the target fish stimulus indicated by the red asterisk.

**Fig. 2.**
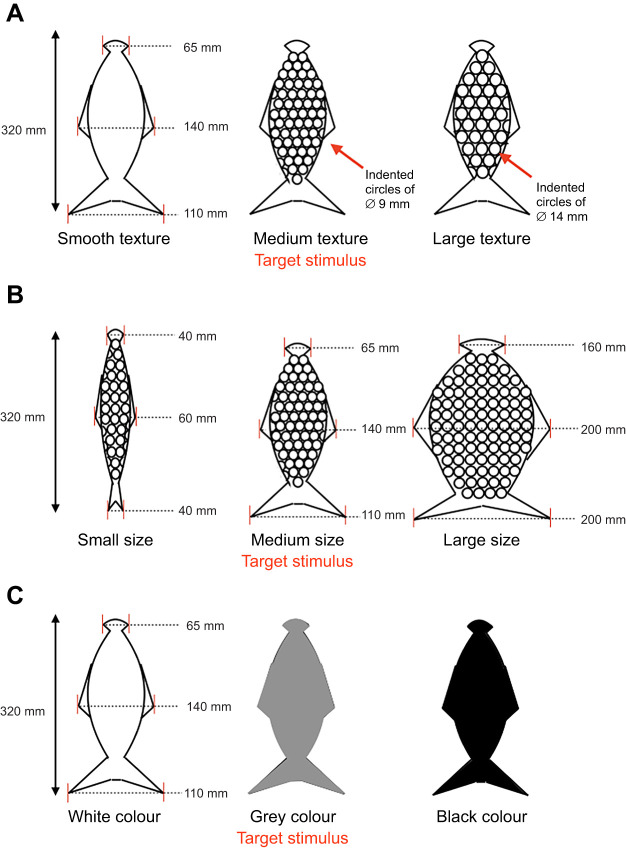
**Fish model stimuli and parameters used for the discrimination tasks.** (A) The texture discrimination task used a smooth distractor fish stimulus, a medium texture target fish stimulus (round indented circles of 9 mm diameter) and a large texture distractor fish stimulus (round indented circles of 14 mm diameter). All stimuli were identical in colour, material, size and shape. (B) The size discrimination task used a small sized distractor fish stimulus (widths of 40 mm head, 60 mm fin and 40 mm tail), a medium sized target fish stimulus (widths of 110 mm head, 140 mm fin and 65 mm tail) and a large sized distractor fish stimulus (widths of 160 mm head, 200 mm fin and 200 mm tail). All stimuli were identical in colour, material and texture. (C) The brightness discrimination task used a white coloured distractor fish stimulus, a grey coloured target fish stimulus and a black coloured distractor fish stimulus. All fish were the same size, shape, texture and material.

For the discrimination tasks, a rig was designed and constructed, consisting of a backboard to attach stimuli and two GoPro (HERO4) video cameras, one on the top and one on the side, filming at 30 frames s^−1^ (Fig. 1). The stimuli were fish shaped (see Fig. 2 for details) and all made using SmoothOn SimpactTM 85A Rubber (SmoothOn distributors Bentley Advanced Materials). The sea lion had to find the target fish-shaped stimulus among two distractor fish-shaped stimuli, for a texture, size or brightness discrimination task (for specific task and set-up details see Supplementary Materials and Methods). The target fish was always the intermediate stimulus – it was always sized at 320×140×50 mm (l×w×d) with widths of 110 mm at the tail, 140 mm at the fin across the body and 65 mm at the head (Fig. 2). For the texture discrimination task (Fig. 2A), stimuli were all the same colour, material, shape and size, only differing in texture: one target stimulus, with a medium texture (round indented circles of 9 mm diameter, with a depth of 4.5 mm, the same texture as inverted bubble wrap); one smooth distractor stimulus; and one large textured distractor stimulus (round indented circles of 14 mm diameter, with a depth of 7 mm, the same texture as inverted large bubble wrap). For the size discrimination task (Fig. 2B), all stimuli had the same medium texture, colour and material with only the size changing: one target stimulus, the same as that used in the texture task; one small-sized distractor stimulus (with widths of 40 mm head, 60 mm fin and 40 mm tail); and one large-sized distractor stimulus (with widths of 160 mm head, 200 mm fin and 200 mm tail). For the brightness discrimination task (Fig. 2C), the fish models all had the same texture (smooth), material, shape and same size, with only the colour varying: one target stimulus, coloured grey; one white distractor stimulus; and one black distractor stimulus. All fish stimuli were attached to J-shaped hooks that rested on three set points on the rig. They were not fixed, so they could be placed and rotated on the rig, following a pseudo-random table as stimulus positions changed order after each trial. The three set positions for the stimuli were indicated on the top of the rig and were equally spaced (160 mm between stimulus J-hooks); however, the stimuli could move somewhat as they were introduced into the water, so the spacing of the stimuli varied slightly (20–40 mm) within trials. In order to accommodate the smaller and large-sized distractor stimulus, the gap between the stimuli was altered, but the gap between the J-hooks remained the same.

### Experimental procedures

Procedures took place within Blackpool Zoos' Active Oceans Arena. All experiments were carried out underwater in the main show pool. The sea lion was blindfolded for both the texture and size discrimination task, so she used her whiskers to tactually discriminate between the different stimuli. The brightness discrimination task was a visual control task. The sea lion was trained using positive reinforcement, so if she successfully identified the target stimulus, she received a whistle signal followed by a fish reward. The sea lion was blindfolded poolside and held by Trainer 1. Once blindfolded, Trainer 2 would position the fish stimuli and submerge the rig underwater. The sea lion was given the ‘find it’ command and released from Trainer 1. The sea lion investigated the stimuli using her whiskers and indicated her response with the following behaviours. Firstly, she rested her nose on the chosen stimulus for a period of time (>3 s), which was quickly followed by moving her whiskers backwards towards the muzzle. The retraction of the whiskers indicated that a choice had been made. For any unsuccessful trials a ‘no’ command was used and the trial reset until the correct response behaviour was given and rewarded. After three unsuccessful trials in succession, the session would be stopped, and the sea lion returned to her pen. The sea lion could undertake up to 100 trials per day. Once all the trials were complete, the sea lion was returned to her pen and released with the rest of the group into the main pool. Two video cameras (GoPro HERO4, 30 frames s^−1^) were used to film the sea lion from the top and the side (Fig. 1; Fig. S2A,B). A total of 30 days of footage was collected for the texture and size discrimination tasks and 20 days of footage for the brightness discrimination task, giving 7200 trials in total (2700 for texture, 2700 for size and 1800 brightness trials).

### Video selection and analysis

All individual trials were then examined to identify clear stimuli interactions from the video footage, where the whiskers and head were clearly in view (specific inclusion criteria are given in Supplementary Materials and Methods). An individual stimulus interaction started from the frame the sea lion whiskers came into contact with any stimulus and ended on the frame prior to the sea lion turning its head away, or the frame prior to the sea lion relaxing her whiskers backwards towards the muzzle (indicating a decision). There were 805 individual stimulus interactions (203 top-down texture, 169 side-on texture; 193 top-down size, 143 side-on size; 75 top-down brightness, 67 side-on brightness) that were tracked manually using the open source Manual Whisker Annotator program (Hewitt et al., 2016). For the top-down camera view, two whiskers (second-most rostral and the second-most caudal whisker) on each side of the face were tracked along with the tip of the nose and a mid-point of the head (Fig. S2B). For the side-on camera view two whiskers (second-most dorsal and second-most ventral whisker) on the right side of the face were tracked along with the mid-point of the head and the tip of the nose (Fig. S2A). Two points were tracked for each whisker: the base of the whisker and a point around two-thirds along the whisker shaft. The tracking was conducted every three frames, which was sufficient for following the head and whiskers.

From the tracked points, nose and whisker measures could then be calculated. Total nose displacement (mm) was calculated by using the nose tracks and measuring the total distance from left to right in the top-down view and up and down, in the side-on view (Fig. S2E,F). The nose distance from the centre of the stimulus (mm) was also calculated as the average left to right distance of the nose tip coordinates from the middle of the stimulus in the top-down camera (Fig. S2F). Whisker angular position (deg) was calculated in both views as the angle between the whisker and the midline of the head, such that forward moving whisker positions (protractions) gave larger whisker angular positions (Fig. S2C,D). It was calculated per whisker and then presented as mean of all whiskers. Whisker amplitude was the difference between the maximum and minimum whisker angular positions; it was also calculated per whisker and then presented as mean of all whiskers (Fig. S2G,H). The time taken to explore each fish stimulus was also calculated in seconds.

### Statistical analysis

As some of the data were not normally distributed, all reported statistics were calculated using non-parametric tests. Main statistical findings are reported in the Results and summary statistics can also be found in Table 1.

**Table 1.**
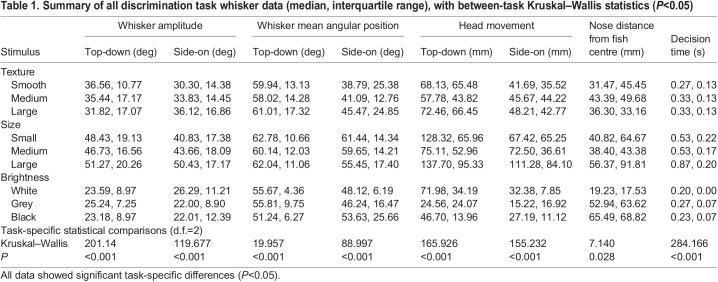
Summary of all discrimination task whisker data (median, interquartile range), with between-task Kruskal–Wallis statistics (*P*<0.05)

## RESULTS

### Task-specific whisker movements

**Fig. 3.**
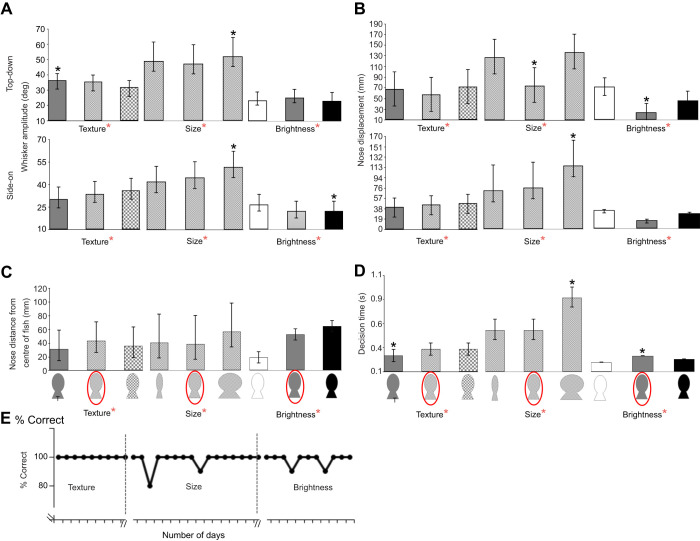
**Summary data from the three discrimination tasks completed by Lo the California sea lion.** (A–C) There were significant differences (*P*<0.05) in whisker (A) and head (nose displacement; B) movements and the nose distance from the centre of the fish stimuli (C) between all tasks. (D) Decision time was significantly greater during the size discrimination task. The longest decision time was associated with the largest fish stimulus. Decision times for the smooth textured fish stimulus were significantly quicker than those for the other textured stimuli. (E) Task performance (percentage correct) showed that Lo achieved 100% correct responses on the majority of days of data collection (post-training); each day included 160–300 individual stimulus interactions. All bar charts show median values with interquartile ranges; the asterisks indicate significant differences (*P*<0.05) between tasks (red asterisks next to the task headings) or between stimuli within the same task (black asterisks above the error bars). The target stimulus for each task is ringed in red.

Lo adopted different whisker and head movement strategies depending on whether she was completing the size, texture or visual brightness discrimination task. She moved her whiskers with larger amplitude (∼74% greater) on the tactile tasks compared with the visual brightness task (Figs 1 and 3). Furthermore, Lo performed significantly larger whisker (47 deg) and head (99 deg) movements during the size task, smaller whisker (34 deg) and head (56 deg) movements during the texture task and very small whisker (24 deg) and head (36 deg) movements on the visual brightness task (reporting average values from top-down and side-on views in Table 1, all *P*<0.05; Fig. 3A,B; Fig. S3). Indeed, during the visual brightness task, head and whisker movements were greatly reduced and Lo usually went straight to the target stimulus using visual guidance (Fig. 1C; Movie 1).

### Task efficiency and performance

Once Lo had learned each task, she successfully identified every stimulus with almost 100% accuracy (Fig. 3E). She was also able to make a decision about each stimulus quickly, with decision times all <1 s (Fig. 3D). Decisions on the size task took the longest to complete, as this task involved exploring over the surface of each stimulus from edge to edge (0.64 s), with the largest stimulus taking the longest time (0.88 s) (Fig. 3D).

## DISCUSSION

These data provide the first evidence to suggest that a California sea lion can make task-specific whisker movements during tactile discrimination tasks. We observed, specifically, that during the size discrimination task, Lo moved her nose and whiskers to the edges of a shape to judge its width (Figs 1B and 3C; Movie 1). Large head and whisker movements around object edges and over object surfaces have previously been documented in size discrimination tasks in walrus (Kastelein, 1988), California sea lion (Dehnhardt and Dücker, 1996) and harbour seals (Grant et al., 2013b), although movements were not specifically measured in these studies. Similarly, humans also feel around the edges of large objects with their fingertips, termed contour-following, to judge size and shape (Gibson, 1962; Klatzky et al., 1987).

During the texture task, Lo made lateral, sweeping movements with her head and whiskers (Fig. 1A; Movie 1), directed around the centre of the stimulus that she was exploring (Fig. 3C). The motion of biological tactile sensors is key to perceiving texture sensations (Diamond, 2010) and is likely to improve the sensation of tactile signals, such as the detection of changes in acceleration and force, which increases sensitivity (Hollins and Risner, 2000; Lederman, 1983). Equivalent stroking, sweeping or rubbing movements to judge textures have been observed by human fingertips (Gibson, 1962; Lamb, 1983; Lederman, 1983) as well mammalian paws in sea otters (Strobel et al., 2018) and squirrel monkeys (Hille et al., 2001). The sweeping of whiskers over surfaces during a texture discrimination task has previously been observed, but not measured, in sea otters (*Enhydra lutris*) (Strobel et al., 2018). Dehnhardt and Dücker (1996) also observed sea lions making lateral head movements, during the tactile examination of different sized and shaped stimuli. This study is the first to measure these whisker sweeping movements in pinnipeds. As in human fingertips, these sweeping movements are likely to be key to discriminating different textures.

The ability to switch whisker exploration strategies between tactile tasks enabled Lo to complete the tasks efficiently with high success. Decision times of around 300 ms on the texture task are similar to observations from sea otter (*Enhydra lutris*) whiskers during a texture discrimination task (Strobel et al., 2018). Indeed, once Lo had learned each task, she successfully identified every stimulus with almost 100% accuracy (Fig. 3E). She also made a decision about each stimulus quickly, with decision times all taking less than 1 s (Fig. 3D). All decision times were much faster in the California sea lion than in squirrel monkeys completing size (3.8 s) and texture discrimination tasks (2.2–5.2 s) with their paws (Hille et al., 2001), as well as human fingertip discrimination studies (76–86 s and 85–94 s, respectively), (Klatzky et al., 1987). However, comparing decision times between studies is challenging as they will be strongly affected by stimulus similarity and prior experience. Nevertheless, quickly and successfully making decisions based on information from whisker signals is likely to be important to pinnipeds, especially during foraging and navigation events in dark underwater environments (Hyvarinen, 1989; Milne and Grant, 2014; Milne et al., 2020).

### Active whisker touch sensing

The ability to adapt sensor movement strategies to different tasks is a key feature of active sensing (Gibson, 1962; Prescott et al., 2011; Wachowiak, 2011; Yang et al., 2016). That Lo uses these strategies to focus on salient features of objects – the texture at the centre of a textured object and the edges of different sized shapes – suggests that the California sea lion whisker system be considered as a truly active touch sensory system. Indeed, we suggest that active control of the whiskers allows California sea lions to efficiently discriminate between different objects. Pinniped whiskers are extremely sensitive (Dehnhardt et al., 1998; Dykes, 1975; Hyvärinen, 1989), with each follicle having 10 times more nerve endings than those of terrestrial mammals (Hyvärinen, 1989). These functional sensitivities are likely to be further enhanced by the execution of the precise movements and strategies described here. This also suggests that the pinniped whisker system incorporates information about touch as well as movement, much like we see in human touch, which integrates inputs from both cutaneous and kinaesthetic receptors (Klatzky et al., 1987). Furthermore, unlike human fingertips, which can decrease sensitivity in cold water, the pinniped whisker system is just as sensitive in cold water temperatures (Dehnhardt et al., 1998). The adaptability of the whisker touch system to perform with high sensitivity both in air and underwater (Dehnhardt and Dücker, 1996; Dehnhardt et al., 1998) means it also has advantages over audition and vision, which tend to be less effective underwater in humans and other mammals.

While pinnipeds have especially long and sensitive whiskers (Dehnhardt et al., 1998; Dykes, 1975; Hyvärinen, 1989; Marshall et al., 2006; Milne et al., 2021), other mammals are also considered to be whisker specialists (Grant and Arkley, 2016; Prescott et al., 2011), especially small, nocturnal, arboreal mammals that actively move their whiskers (Grant et al., 2018; Muchlinski et al., 2020). As whisker movements are found across many orders of mammals (Muchlinski et al., 2020) and their muscle architecture is highly conserved (Grant et al., 2013a; Grant et al., 2017; Muchlinski et al., 2013), this suggests that other mammals may well engage in task-specific whisker movements.

### Limitations of the study

Although Lo was blindfolded, she did not wear earphones. Earphones have previously been employed to remove auditory cues during experiments (Grant et al., 2017; Krüger et al., 2018). While some auditory cues might have been perceived during the rotation of the stimulus changeover, this appears unlikely as the sea lion did not go straight to the target fish in the texture and size discrimination tasks (as she did during the visual task) but felt many of the fish models with her whiskers. Previous studies have also suggested that over-training of tasks might affect whisker movements (Dehnhardt and Kaminski, 1995; Grant et al., 2013a,b), where the animal might choose the most efficient way to undertake the task, rather than making natural whisker movements. It may also be that the sea lion is remembering one stimulus rather than making a true comparison. Developing more natural tests, such as incorporating tactile exploration during food a preference task, may encourage more natural whisker movements that would not require training. The ideal scenario would be to film wild animals making decisions about live prey items based on tactile information, but this would be challenging, both experimentally and ethically.

It is also difficult to access large numbers of trained marine mammals (Dehnhardt and Dücker, 1996; Wieskotten et al., 2010a, b; Grant et al., 2013a,b); therefore, it is common to only use one individual (Dehnhardt and Dücker, 1996; Wieskotten et al., 2010a, b). Although we started training four sea lions, only one, Lo, managed to reach the threshold required for data collection. We can clearly see that Lo employs different head and whisker movement strategies between the tasks (Figs 1 and 3). We also reviewed the collected recorded footage from the other three sea lions by eye and found that these individuals made similar head and whiskers movements to Lo. Specifically, we observed that during the texture discrimination task, the other three sea lions (Rubi, Gala and Filipa) also made sweeping movements with their whiskers and positioned their head to the centre of the stimuli. Furthermore, in the size discrimination task, Rubi and Gala focused their nose and whiskers more towards the edges of each stimulus and spent more time investigating the larger stimulus. Dehnhardt and Dücker (1996) have also previously documented that whiskers move around object edges and over object surfaces in size discrimination tasks in California sea lions. Therefore, we suggest that the strategies we observed in Lo are likely to be adopted by other California sea lions too.

As the stimuli were made using a silicon mould, they contained some imperfections. Some wear and tear of the stimuli also occurred during training and exposure to salt water. However, the main differences between the stimuli were always those that were designed for the individual discrimination task, i.e. texture, size and brightness, rather than any other imperfections. The size discrimination task also had differences in stimulus shape, which reflected the inherent nature of that task. Therefore, this task might be representative of both size and shape discriminations. Imperfections in the stimuli may also have caused the brightness task to be multi-sensory as there were also likely to be slight tactile differences between stimuli. However, the sea lion went straight towards the target stimuli in the brightness task; therefore, visual guidance appeared to be the prominent sense employed in this task. Despite variation in the stimulus parameters, we are confident that this study provides the preliminary evidence to show that task-specific whisker movements are employed in California sea lions.

### Future recommendations

The ease of training many mammalian species (Arkley et al., 2014; Milne and Grant, 2014) and tracking whisker movements (Gillespie et al., 2019; Hewitt et al., 2016; Petersen et al., 2020) means that the whisker system is an excellent model to explore hypotheses in active sensing. Identifying whisker movement strategies during tactile object exploration furthers our understanding of mammalian sensing and perception. Applying these specific movement strategies to artificial sensory systems will also help progress research on tactile robotic control and performance (Luo et al., 2017; Pearson and Salman, 2019; Roberts, 1990). The next step will be to examine these movement strategies in more natural settings to assess how information from the whiskers may mediate complex behaviours and survival strategies in pinnipeds, such as during foraging and prey capture. This study provides an initial basis from which to further explore the phenomenon of active touch sensing. Studies of this kind can be applied not only to pinnipeds but also to other mammals.

## Supplementary Material

Supplementary information
