# Supplementary material for: California sea lions employ task-specific strategies for active touch sensing
Source: J Exp Biol. 2021 Nov 5;224(21):jeb243085. doi: 10.1242/jeb.243085 (PMC8627572; doi:10.1242/jeb.243085)
Supplement: Supplementary information [file jexbio-224-243085-s1.pdf]

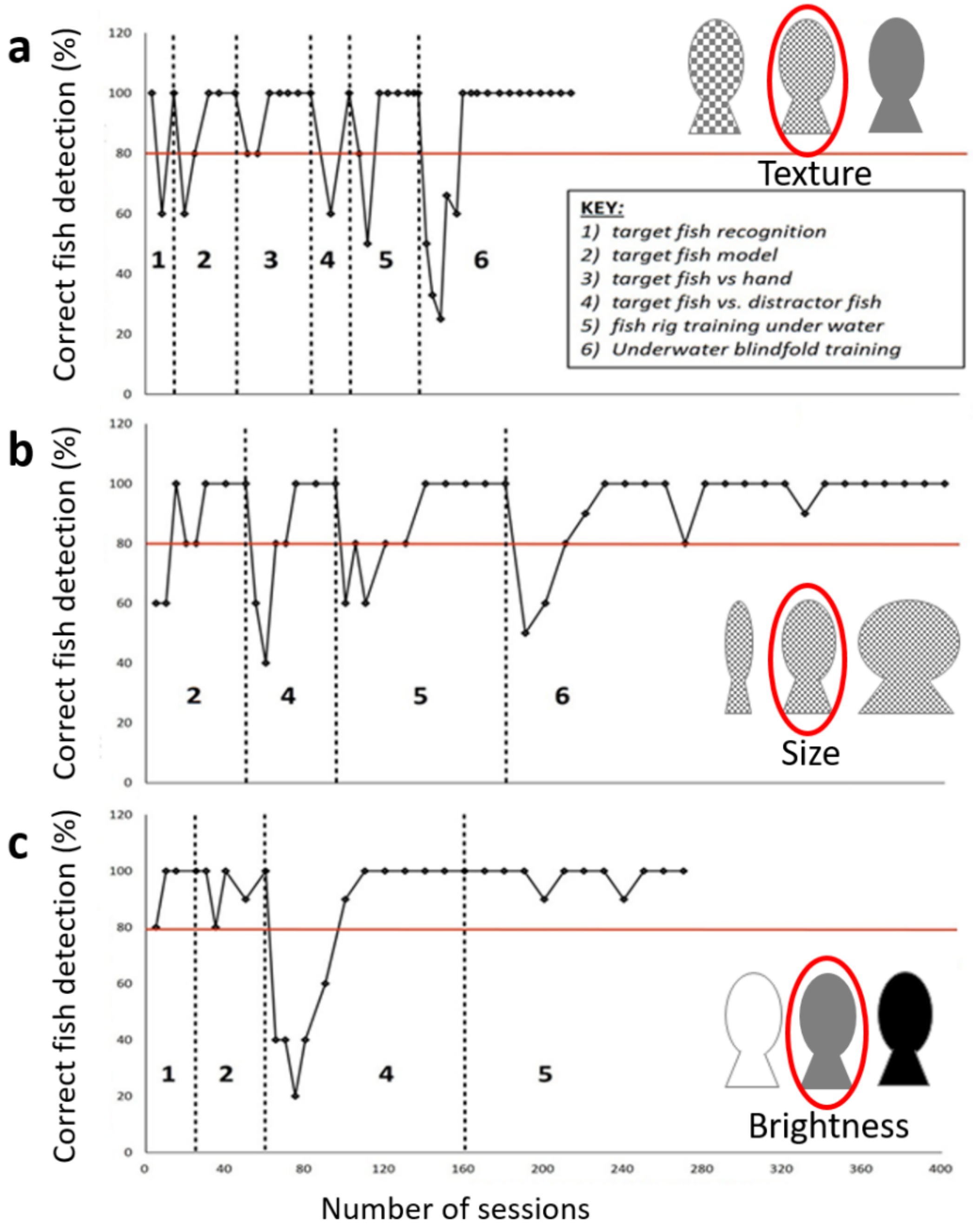

Fig. S1. Training. One female California sea lion (Lo, aged 15 years) completed all aspects of the training and reached the threshold required for data collec=on in each task. Four California sea lions were originally trained for the study; however, during training two were moved to another collec=on with one having a prominent right-hand bias and one refused to wear the blindfold, so did not perform the task to an appropriate threshold level. Lo's training curves for each task are shown. Training during the tasks with a learning criterion set as >80% correct, for three consecutive sessions, during the texture (**a**) , size (**b**) and brightness (**c**) discrimination tasks, for the following stages: 1) target fish recognition, where the sea lion was introduced to target on the target fish stimulus; 2) target fish, where the sea lion was trained to target on to the target fish stimulus as it was moved around; 3) target fish vs. hand, where the sea lion was trained to distinguish the target fish from t trainer's hand; 4) target fish vs. distractor fish training, where the sea lion was trained to distinguish the target fish from the distractor fish; 5) fish rig training under water, where the sea lion was trained to do the task underwater without a blindfold; 6) underwater blindfold, where the sea lion was trained to complete the task underwater with a blindfold. Not all training stages were needed in each task, since the sea lion had previously learned them.

Supplementary Materials and Methods

Experimental apparatus and tracking

Experimental Procedures

Sessions occurred during the day over the following time frame: for the texture discrimination task three months throughout May, June and July 2017; for the size discrimination task three months during November, December and January 2017-2018; and finally for the brightness discrimination task, sampling took place over two months in February and March 2019. No significant alterations in Lo's whisker length took place between tasks (compare whisker lengths in example footage in Fig. 1). Pilot studies of each of the full discrimination tasks took place over three days prior to data collection to make sure the sea lion was fully desensitised to the experimental procedure, the apparatus and to check the positioning of the camera for whisker detection.

During the task, the same trainers were present on each occasion. The sea lion was blindfolded for both the texture and size discrimination task, but not for the brightness discrimination task. The sea lion was trained using positive reinforcement, so if she successfully completed a trial, she received a fish reward. During a session, the sea lion received approximately 20% of her daily food amount. This was freshly thawed cut Atlantic Mackerel (Scomber scombrus), Atlantic Herring (Clupea harengus), or whole Capelin (Mallotus villosus) and European Sprat (Sprattus sprattus). A total of 30 days of footage was collected for the texture and size tasks and 20 days of footage for the visual brightness discrimination task (brightness). This gave 7200 trials (2700 for texture, 2700 for size and 1800 brightness trials).

Video selection and analysis

The GoPro Studio 2.0 programme (https://gopro-studio.en.softonic.com/) was used to remove the fisheye effect from the footage prior to video analysis. All trials were then examined to see if the video met the inclusion criteria for tracking: (i) all whiskers on both sides were visible for the Top-down Camera and all whiskers on one side were visible for the Side-on Camera, from approach to contact with the stimuli; (ii) the sea lion did not pre-emptively choose the target before the rig was placed in the water, (iii) the sea lion gave the correct answer. After viewing all the video footage, this gave a total of 805 individual interactions with one of the stimuli; 372 for the texture discrimination task (203 on the top-down camera and 169 on the side-on camera), 336 for the size discrimination task (193 on the top-down camera and 143 on the side-on camera) and 142 for the brightness discrimination task (75 on the top-down camera and 67 on the side-on camera). The number of stimulus interactions for the visual brightness task was lower due to the sea lion rarely exploring the distractor stimuli. Details of tracking and statistical analyses can be found in the main manuscript text.

Journal of Experimental Biology • Supplementary information

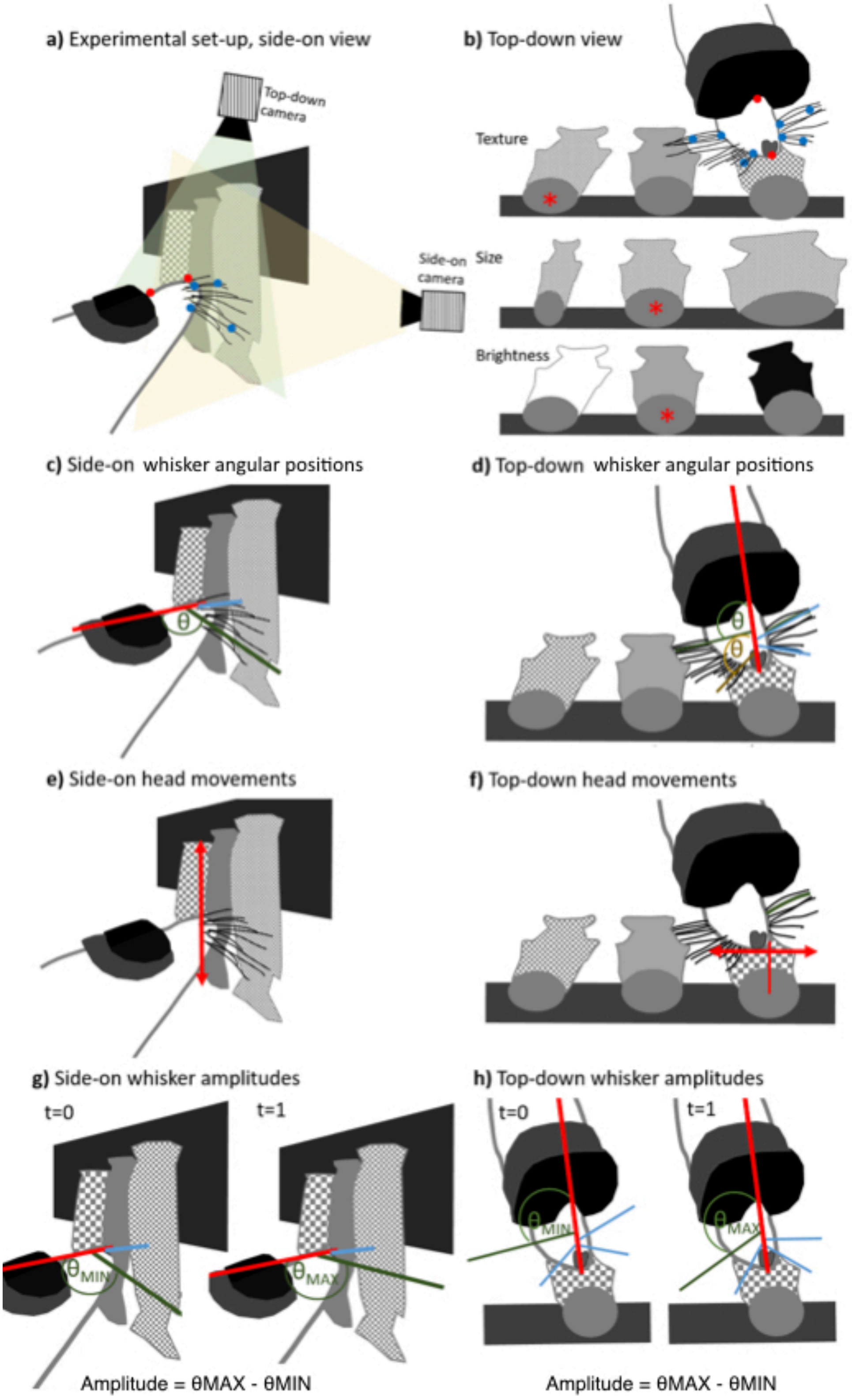
a) Experimental set-up, side-on view
Top-down camera
Side-on camera
b) Top-down view
Texture
Size
Brightness
c) Side-on whisker angular positions
θ
d) Top-down whisker angular positions
θ
θ
e) Side-on head movements
f) Top-down head movements
g) Side-on whisker amplitudes
t=0
t=1
θMIN
θMAX
Amplitude = θMAX - θMIN
h) Top-down whisker amplitudes
t=0
t=1
θMIN
θMAX
Amplitude = θMAX - θMIN

Fig. S2. Extended methods figure. Experimental set-up in the side-on (**a**) and top- down (**b**) view. The head (red points) and whiskers (blue points) were tracked from the video footage in two views as the sea lion explored each stimulus. Stimuli varied between the texture, size and visual brightness discrimination tasks, the target stimulus for each task is indicated by the red asterisk (*). Whisker angular positions were extracted from the tracking by calculating the angle (θ) that the whiskers made with the midline of the head in both the side-on (**c**) and top-down views (**d**). Examples indicated here are for the right hand ventral whisker angle in the side-on view (green in panel c); and in the top-down view, the rostral whisker angle (yellow in panel d) and caudal whisker angles (green in panel d). Tracked whiskers in the top-down view included two rostral and two caudal whiskers (blue lines) from each side. Head movements were calculated during the stimuli exploration in the side-on (**g**) and top-down view (**h**). Nose distance from the fish center was also calculated in the top-down view (panel f). Whisker amplitudes were calculated as the difference between the maximum whisker angular position (green line θMAX at t=0) and the minimum whisker angular position (green line θMIN at t=1) i.e. the difference between the most backward and most forward angular position of that particular whisker within that individual stimulus interaction.

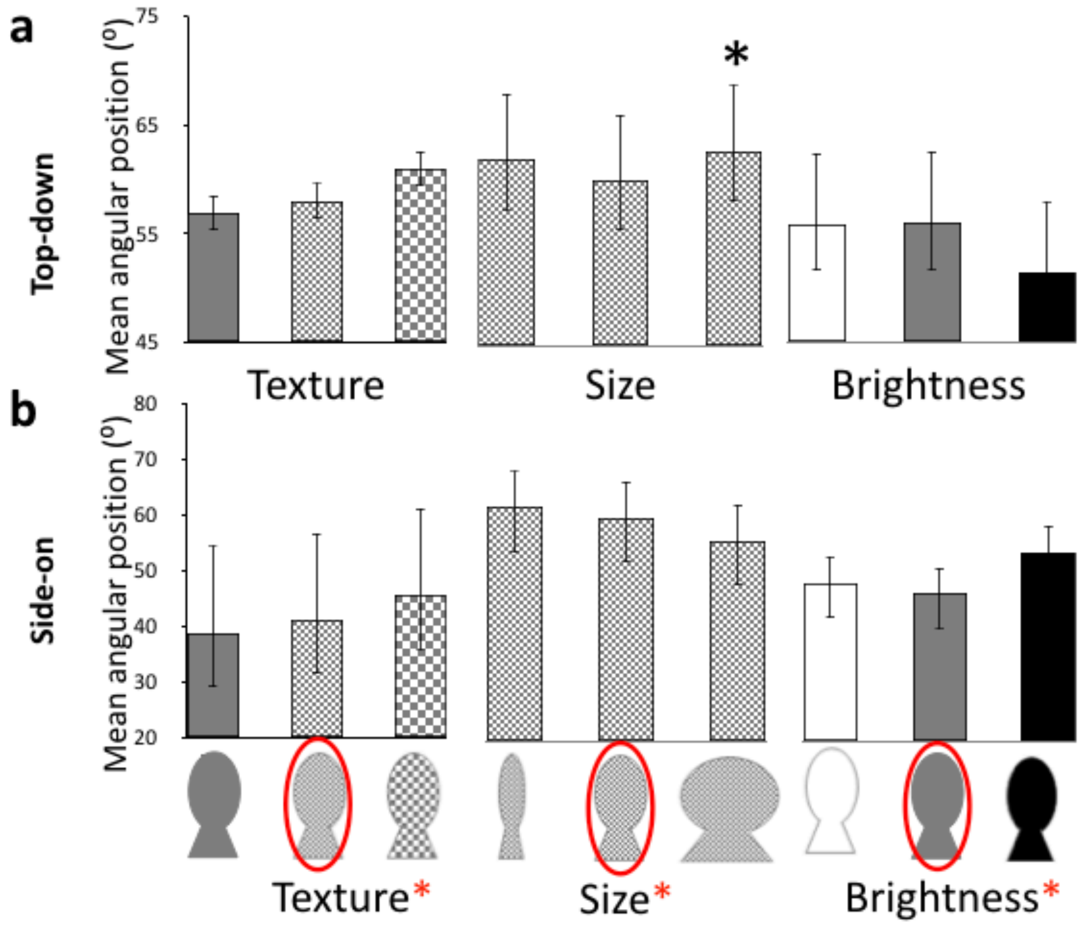

Fig. S3. Supplementary results for mean angular position from the three discrimination tasks. All graphs show median values with interquartile ranges, and the asterisks (*) indicate significant differences ($p<0.05$) of tasks (red asterisks, next to the task headings) or stimuli, compared to other stimuli within the same task (black asterisk, above the error bars). There were significant differences ($p<0.05$) between all the tasks for mean angular position in the side-on view, but not the top-down view.

Journal of Experimental Biology • Supplementary information

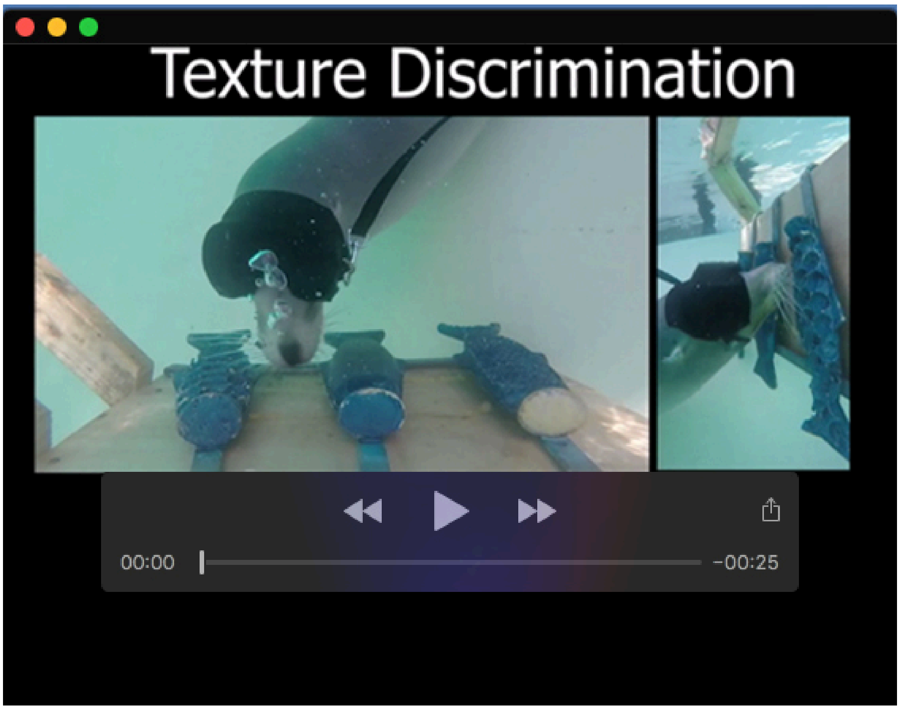

Movie 1. Lo the California sea lion completing each of the three discrimination tasks. Texture Discrimination Task: During the texture task, Lo made lateral, sweeping movements with her head and whiskers; Size Discrimination Task: During the size discrimination task Lo moved her nose and whiskers to the edges of a shape to judge its width; Brightness Discrimination Task: During the visual brightness task, head and whisker movements were greatly reduced and Lo usually went straight to the target stimulus using visual guidance.
